# Animal science program accreditation? This is the future

**DOI:** 10.1093/af/vfaa026

**Published:** 2020-07-23

**Authors:** Margaret E Benson, Wesley N Osburn, Marc Bauer, Glenn C Duff, Nancy A Irlbeck, Mike L Looper, Tom A Rathje

**Affiliations:** 1 Department of Animal Sciences, Washington State University, Pullman, WA; 2 Department of Animal Science, Texas A&M University, College Station, TX; 3 ||Department of Animal Sciences, North Dakota State University, Fargo, ND; 4 $Clayton Livestock Research Center, New Mexico State University, Clayton, NM; 5 ¶Department of Animal Science, University of Arkansas, Fayetteville, AR; 6 *DNA Genetics, Columbus, NE

**Keywords:** accreditation, educational value, programs, quality

**Implications**

- The American Society of Animal Science is in a strong position to support and implement a peer-driven, comprehensive voluntary accreditation program based on its large and diverse animal science professional membership and its involvement with academic programs training future animal science graduates.
- Students, employers, academic institutions, and potential employers benefit from accreditation by ensuring that animal science educational programs meet or exceed academic standards defined by animal science professionals.

## Introduction

Should voluntary accreditation of 4-year animal science degree programs be the future?

The American Society of Animal Science (ASAS) Committee on Accreditation suggests it is needed and yes, it is the future. Higher education continues to evolve with new challenges, opportunities, and expectations. Graduates with 4-year degrees in Animal Science have been rewarded with plentiful and diverse career options and opportunities. However, now is not the time to rest on our laurels. As times change, so should animal science programs to continually meet current and future implications in our educational processes. Those who invest time and resources in undergraduate animal science programs (students, parents, and employers) increasingly expect more accountability and documentation of what they can expect from their investment. Accreditation is a quality assurance program already used by many undergraduate disciplines including those in agriculture. Now is the time for animal science programs to proactively offer this value-added asset to their animal science degree programs. It is essential to be proactive and take responsibility for our future, for if we do not, others with less commitment may make the decisions for us.

ASAS is committed to a strong future for the next generation of animal scientists. With a large and diverse membership of professional animal scientists, it is well positioned to lead an accreditation effort. ASAS has accepted the responsibility of implementing a voluntary Accreditation Program that will be available initially to institutions within the United States awarding 4-year Bachelor of Science degrees in Animal Science. The work of ASAS is guided by five core principles (https://www.asas.org/about/history-and-mission) and two of those core principles directly align with accreditation; specifically principle #4 *Career development for animal scientists, educators and producers is essential to the viability of the allied and animal industries*; and #5 *Animal science and the production of animal-sourced foods must continually evolve to meet the needs and values of society*. The accreditation process requires departments to conduct a self-assessment of their animal science undergraduate program based on academic standards established by an accrediting body/agency. Data generated by this self-assessment is reviewed by trained third-party academic professionals who have received graduate degrees in animal science or animal science related disciplines. Areas that are reviewed range from reviewing curriculum and learning outcomes to the resources available to support students’ academic experience (i.e., advising support, animals, and facilities). Compliance with established accreditation standards results in the animal science program becoming accredited.

## ASAS Approach to Accreditation

Development of an accreditation option for animal science programs began several years ago and in 2014 the ASAS Board of Directors voted to establish standards for accreditation of Animal Science programs. Since then, there have been multiple actions and votes by the ASAS Board of Directors to investigate and implement the process. To date, the ASAS Board of Directors has voted to charge the Animal Science Accreditation Committee with researching, developing, and proposing an implementation plan, and they have voted to enact and provide infrastructure for an accreditation program for undergraduate programs. The proposed Animal Science Standards for Accreditation were posted for member comment and an implementation strategy was forwarded to the ASAS Board of Directors.

The Animal Science Accreditation Committee, composed of academic and industry professionals, developed an accreditation process, and established proposed standards for accrediting Animal Science undergraduate programs. This committee investigated other accreditation programs currently offered in a variety of disciplines. Examples of professional societies leading accreditation programs include but are not limited to disciplines of range management, forestry, and landscape architecture. The Society of American Foresters (SAF) is responsible for accreditation in that discipline (https://www.eforester.org/Main/Certification_Education/Accreditation/Main/Accreditation/Accreditation_Home.aspx?hkey=acede682-0ce7-4202-85e6-e3371eb38cdc). Volunteers from SAF academic and industry membership make their accreditation process possible by overseeing the approved standards and reviewing academic programs. Forestry programs volunteer to undergo a comprehensive review by SAF periodically to determine if the criteria set by their society leaders are met. Accredited range management programs are approved by the Society of Range Management and as a part of its mission has accredited range management programs since 1977 (http://rangelands.org/srm-academic-resources/universities-colleges/). The Landscape Architecture Accreditation Board (LAAB) develops and enacts accreditation standards and processes authorized by the American Society of Landscape Architecture board of trustees (https://www.asla.org/aboutlaab.aspx). The accreditation of these programs assures a quality and professional education for enrolled students, and provides confidence to all invested.

Veterinary education is accredited by the American Veterinary Medical Association (AVMA) and is responsible for accrediting both Veterinary Schools and Veterinary Technology Programs (https://www.avma.org/education/center-for-veterinary-accreditation). The veterinary discipline impacts many animal science undergraduates and the AVMA relies heavily on accreditation as a tool to identify programs with a commitment to quality and continuous improvement provided by peer review.

Using this information, the ASAS Accreditation Committee developed the following objectives of Animal Science Program Accreditation:

- To improve the overall quality of animal science education through program self-evaluation and peer review by qualified academic and industry professionals.
- To foster excellence in animal science educational programs through the periodic revision of standards for accreditation and to apply those standards in evaluation of the educational environment and effectiveness of animal science programs.
- To recognize the diversity of animal science programs that in addition to production animal agriculture, may include care of companion animals, conservation of species (e.g., captive exotics), and management of laboratory animals.
- To assure students, employers, the public, and other organizations that accredited animal science programs have educational objectives and outcomes consistent with current professional standards for the field of animal science, and have adequate resources to accomplish these objectives.

**Figure 1.**
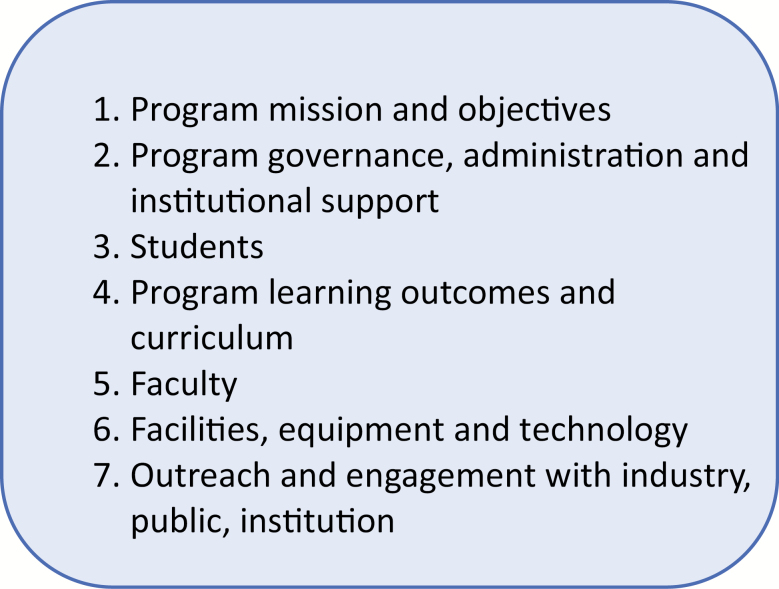
The components of proposed Animal Science Accreditation Standards.

Accreditation standards for seven components of an animal science undergraduate program were developed and recently published for ASAS membership review and comment. The components of the standards are identified in Figure 1.

To implement the accreditation process, the Accreditation Committee proposed the formation of the Animal Science Council on Accreditation. This council will be composed of professional ASAS members who have the authority to enact the accreditation process for programs who seek accreditation, identify accreditation review teams, deliberate on the institutional application, and review team findings and render a decision on whether an animal science program has met established accreditation standards to receive program accreditation.

**Figure 2.**
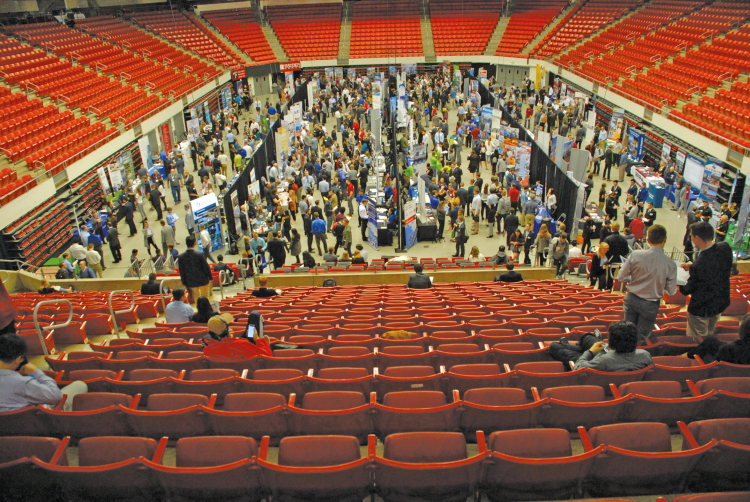
Students interact with industry employers during a career fair. Photo by: Tyler Barstow, Washington State University, Career & Employer Relations Liaison.

Accreditation of animal science programs signifies that professionals in the animal science disciplines are proactively committed to high-quality education and training of the next generation of animal scientists; and that required standards have been identified for their programs. When the Animal Science Standards for Accreditation are met, it ensures that the curriculum covers essential animal science concepts; students acquire knowledge and experiences within the animal science disciplines; that the accredited program has the appropriate institutional support enabling a sustained high-quality program to be delivered to students; and that the program provides the education needed by graduates of the program to step into diverse professions of animal science, fully prepared to meet industry needs. Students learn of career opportunities and how to prepare for these careers in a variety of ways including career fairs where students interact directly with employers (Figure 2).

## Benefits of Accreditation

Accreditation of professional programs, through program self-evaluation and a third-party peer review processes, is a long proven and accepted method of ensuring quality and consistency of academic programs and is widely used throughout academic disciplines. Peer review of individual agriculture departments including animal science is nothing new and was a routine periodic process provided by the United States Department of Agriculture National Institute of Food and Agriculture. This review option no longer exists. Recognizing that academic reviews are critical to demonstrate that animal programs are meeting the needs of our students, stakeholders, and society as a whole, accreditation is an option that fills that need.

**Figure 3.**
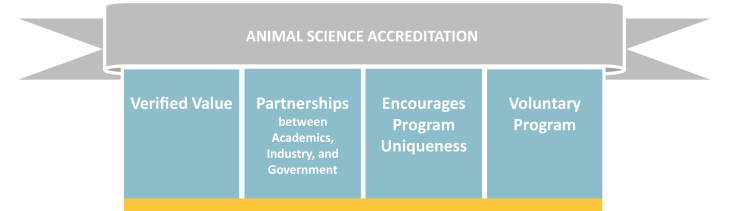
Important characteristics of animal science accreditation.

Accreditation of animal science programs will be of value to the academic departments, colleges, and institutions awarding the degrees, to the students graduating from these programs and to the employers of graduates from these programs. The accreditation process engages animal science professionals from academia, industry, and government to provide objective external review and attest to program quality and commitment to continuous improvement. With increasing expectations of accountability in higher education, accreditation provides a valued opportunity to verify program quality and capacity for training students who are well prepared to enter diverse professional positions upon graduation or continue their education in professional degree programs. Figure 3 summarizes what accreditation of animal science programs can offer.

## Why Many Believe This Is an Important Initiative for ASAS to Provide

Not everyone agrees that animal science programs need an accreditation option, nor that ASAS should get involved in accrediting animal science programs. However, after several years of input from membership and all sectors of the diverse profession, most agree that this voluntary program is, in fact, needed and perhaps now more than ever in a challenging environment where funding is limited. ASAS is uniquely positioned to provide the leadership for and support of this effort.

Three important characteristics that make Accreditation an asset for those who want it:

1. Verified value
  The value of a college education is of interest to all investors in that education. This includes the students, their parents, prospective employers of the graduates, and the academies delivering the education. Value is measured and perceived differently by everyone. Programmatic accreditation provides a common process used throughout academia to ensure defined standards of quality that are met by the program and degree granting institution. Most degree granting programs are housed at institutions already engaged in regional or national accreditation which accredits the entire institution. Programmatic accreditation is a status earned by an animal science program.
  Currently students and parents of students have access to little subjective information on what can be expected from an animal science program and how it measures up to industry and employer expectations. Prospective employers have asked for an understanding of the content and training they can expect animal science graduates to have experienced during their undergraduate career. Accreditation provides public acknowledgment and confidence that quality of a specific program meets standards as verified by a third party of professionals within that program area.
  Institutions are committed to offering outstanding undergraduate experiences and training. Those voluntarily seeking accreditation welcome the opportunity to have their programs reviewed by animal science professionals, benefit from the input and critique received, and have yet another means of demonstrating the value of their program to students, academia, industry, and society. Accreditation also supports and enhances institutional assessment programs already in place.
2. Partnerships are valued and necessary
  The diverse disciplinary expertise of animal science is known to engage academic, industry, and government partners in teaching, research, and extension activities. These partnerships are an essential element in past, present, and future advances and accomplishments in the field. Therefore, Animal Science accreditation relies on an active engagement and participation by academic, industry, and government partners in the accreditation process. This is necessary to ensure that the next generation of animal scientists are well prepared and trained. Input by industry professionals provides essential and timely insight into the ever-changing arena in which animal scientists work. Incorporation and application of new technologies, discoveries, and regulations must be considered as the field continues to evolve. Many of the careers of future animal scientists begin with their employment in these very career paths. Accreditation values and uses the contributions of all animal science professional partners in preparing for the future.
3. Program uniqueness is encouraged
  Animal Science programs offering 4-year Bachelor of Science degrees in animal science vary significantly in size, scope, geographic location, and focus. Individuality of programs is encouraged with the proposed accreditation program. Accreditation standards are not written to be prescriptive in their requirements but rather encourage programs to employ novel, innovative, and evolving methods and processes to accomplish the required standards. The proposed standards identify key required elements of an accredited program but do NOT identify how those elements might be accomplished. It is by intent that the WHAT of the requirements are defined but the HOW is determined by the institution/department applying for accreditation. Accreditation is not designed to make all programs look alike, teach alike, serve the same student demographics, or serve the same student professional aspirations. Large enrollment programs will manage courses and course offerings differently than those with smaller enrollments. Programs training mainly preprofessional students (e.g., Graduate School and Veterinary School) will differ from those with students intending to seek industry or production careers immediately upon graduation. Programs will vary in how they provide students with experiential learning with animals and other experiential learning options.
  Uniqueness of individual programs is expected and encouraged. While the standards identify key elements that all programs must possess, it is up to the program administration to show how those requirements are met. The success of a program meeting those standards is evaluated by the visiting review team and self-study documentation.

## Conclusion

The accreditation process engages animal science professionals from academia, industry, and government agencies to provide objective external review and provides data to assess program adherence to academic standards and commitment to continuous improvement. With increasing expectations of accountability in higher education, accreditation provides a valued opportunity to verify program quality and capacity for training students who are well prepared to enter diverse professional positions upon graduation, or continue their education in professional degree programs. ASAS is in a strong position to support and implement a peer-driven, robust, and comprehensive accreditation program. The membership and board of ASAS represents the diversity of professionals working in the animal sciences and working within the programs producing today’s graduates. As the pressure for accreditation grows from a range of related organizations, ASAS is the logical entity to administer and provide leadership to develop and implement an accreditation process based on standards widely accepted and supported by its membership. Recognition of these standards, and support of ASAS in this role, will provide a peer-created, peer-driven, widely accepted accreditation program that will establish the standards and a third-party validation of all of our efforts to educate and train the next generation of animal scientists and industry professionals.

## About the Authors

**Margaret E. Benson** is Professor Emeritus, in the Department of Animal Science at Washington State University, Pullman, WA. She serves as the Chair of the ASAS Accreditation Committee. Dr Benson was Chair of the Department of Animal Science for approximately 10 years and prior to that spent most of her professional career at Michigan State University where she was engaged in research, teaching, Extension and administration. She received her PhD in Ruminant Nutrition from the University of Kentucky and BS and MS degrees in Animal Science from North Dakota State University. Dr Benson is a Fellow in ASAS and was ASAS President.

**Wesley N. Osburn** is an Associate Professor at Texas A&M University in the Department of Animal Science. His outreach efforts include assisting the meat and poultry industry with processing systems troubleshooting and conducting value added processed meat workshops. His teaching interests include value added processing, food safety, quality assurance, and team problem solving. Dr Osburn’s research focuses on processing technologies to improve meat protein functionality, processing aid solutions to enhance meat product shelf life and innovative meat curing systems. Dr Osburn received both his BSc and MSc degrees from Texas A&M University and his PhD from the University of Nebraska-Lincoln.

**Marc Bauer** is associate professor and interim head of the Department of Animal Sciences at North Dakota State University. In 1996, he obtained a PhD from the University of Kentucky in digestive physiology and has been at North Dakota State University since. He served as the undergraduate curriculum coordinator for the department. His research interests are in beef cattle nutrition and intake.

**Glenn C. Duff** is a Professor and Superintendent at the New Mexico State University Clayton Livestock Research Center in Clayton, New Mexico. He earned his PhD in animal nutrition from New Mexico State University, a MS from the University of Arkansas, and BS degree in Animal and Dairy Sciences from Northwest Missouri State University. His research program focuses on health and performance of newly received, highly stressed beef calves. He also works on performance during the finishing period in beef cattle and performance during the growing period. His work also includes health and performance of neonatal dairy calves.

**Nancy A. Irlbeck** is currently on faculty at Washington State University in the Department of Animal Sciences as a Comparative Nutritionist. She is in a primarily teaching role as a Clinical Associate Professor after retiring from Colorado State University after 27 years. Dr Irlbeck holds Emeritus positions from CSU as an Associate Professor of Animal Sciences and as Associate Dean of Academic Affairs from the College of Agricultural Sciences. Dr Irlbeck has Bachelor of Science and Master of Science degrees in Animal Sciences from Iowa State University and a PhD in Ruminant Nutrition from the University of Nebraska-Lincoln.

**Michael L. Looper** is Professor and Head of the Department of Animal Science at the University of Arkansas. He coordinates research, teaching, and extension programs of 22 faculty members. Prior employment included work at New Mexico State University and USDA-Agriculture Research Service. He received his BS and MS from the University of Arkansas and his PhD at Oklahoma State University. Looper was named the USDA-ARS, Herbert L. Rothbart Outstanding Early Career Research Scientist, and received the Presidential Early Career Award for Scientists and Engineers. Looper is an ASAS Fellow, and has served as a Director-at-Large and President of ASAS.

**Tom A. Rathje** serves as Chief Technical Officer at DNA Genetics LLC. Tom currently works with key accounts and is responsible for genetic and product research including ongoing strategy for the genetic program, genomic technologies, trait development, nutrition, reproductive technologies, and production research. Tom received his PhD and MBA from the University of Nebraska-Lincoln.

